# NON-COVID-19 UPPER RESPIRATORY INFECTION DECREASES DURING THE COVID-19 PANDEMIC

**DOI:** 10.1093/geroni/igad104.2267

**Published:** 2023-12-21

**Authors:** Alec Pruchnicki

**Affiliations:** Vista on 5. th ALF, New York City, New York, United States

## Abstract

During the Covid 19 pandemic, the Vista on 5th Assisted Living Facility in New York City instituted a wide variety of pharmacological and non-pharmacological interventions (NPIs) to control the spread of the disease. Before and after vaccines were available for Covid 19, this combination of approaches reduced the usual seasonal variation of non-Covid upper respiratory infections (URIs). Almost all residents of the 127-bed facility were seen by the on-site physician with the onset of URI symptoms. Almost all those with symptoms were give an anti-histamine and cough syrup for relief. The single off-site pharmacy which supplied these medications tabulated the total amount of medications dispensed over the four years of 2019 to 2022. During December of 2019, normally the peak month for URIs at the facility, and the last month before significant Covid infections occurred, 67 prescriptions of these two types were dispensed. During 2020 NPIs and these medications kept new Covid 19 infections to about one dozen over the year. Medications being dispensed for all URIs in December of 2020 decreased by 69% to 21. Similar low rates of URI symptoms were seen in December 2021 and 2022 with only 18 prescriptions written in both months. Although some variation from month to month occurred because of other syndromes, the overall burden of URIs during the normally peak respiratory infection season was greatly reduced probably from the strict imposition of NPIs.

